# An Efficient and Privacy-Preserving Scheme for Disease Prediction in Modern Healthcare Systems

**DOI:** 10.3390/s22155574

**Published:** 2022-07-26

**Authors:** Shynu Padinjappurathu Gopalan, Chiranji Lal Chowdhary, Celestine Iwendi, Muhammad Awais Farid, Lakshmana Kumar Ramasamy

**Affiliations:** 1 School of Information Technology and Engineering, Vellore Institute of Technology, Vellore 632014, India; pgshynu@vit.ac.in (S.P.G.); chiranji.lal@vit.ac.in (C.L.C.); 2 School of Creative Technologies, University of Bolton, Bolton BL3 5AB, UK; 3 Centre of Excellence for AI and ML, Hindusthan College of Engineering and Technology, Coimbatore 641050, India; research.laksha@gmail.com

**Keywords:** Internet of Things, authentication, secure data transfer, disease prediction system (DPS), substitution cipher, log of round value-based elliptic curve cryptography (LR-ECC), Gaussian Kernel-based linear discriminant analysis (GK-LDA), elephant herding genetic algorithm-based deep learning neural network (EHGA-DLNN)

## Abstract

With the Internet of Things (IoT), mobile healthcare applications can now offer a variety of dimensionalities and online services. Disease Prediction Systems (DPS) increase the speed and accuracy of diagnosis, improving the quality of healthcare services. However, privacy is garnering an increasing amount of attention these days, especially concerning personal healthcare data, which are sensitive. There are a variety of prevailing privacy preservation techniques for disease prediction that are rendered. Nonetheless, there is a chance of medical users being affected by numerous disparate diseases. Therefore, it is vital to consider multi-label instances, which might decrease the accuracy. Thus, this paper proposes an efficient privacy-preserving (PP) scheme for patient healthcare data collected from IoT devices aimed at disease prediction in the modern Health Care System (HCS). The proposed system utilizes the Log of Round value-based Elliptic Curve Cryptography (LR-ECC) to enhance the security level during data transfer after the initial authentication phase. The authorized healthcare staff can securely download the patient data on the hospital side. Utilizing the Herding Genetic Algorithm-based Deep Learning Neural Network (EHGA-DLNN) can test these data with the trained system to predict the diseases. The experimental results demonstrate that the proposed approach improves prediction accuracy, privacy, and security compared to the existing methods.

## 1. Introduction

The current healthcare system (HCS) is an intricate data-driven network that depends on continuous monitoring of patients, data sharing, and streaming [1,2]. It utilizes advanced big data analytics to offer necessary health services to patients [3,4,5]. While offering high-quality treatment to in jeopardy patients, a solution is necessary to lower the pressure on HCS [6]. The growing technology, which is expected to support an extensive range of healthcare apps, is the wearable monitoring system and the IoT [7,8,9]. Doctors recommended that people utilize various varieties of IoT-centered products, which are competent in displaying and storing distinct types of pathological data concerning various diseases [10,11,12].

Nevertheless, wearable gadgets for healthcare problems [13], namely smart ECG machines, Bluetooth blood glucose measuring devices, and 3G BP measuring devices, could be utilized to monitor blood sugar, ECG, and blood pressure along with various physiologic symptoms [14,15,16]. DPS plays a primary role in peoples’ lives, and it is pondered as an important topic by various academics [17,18]. Here, Artificial Neural Networks (ANN), Principal Component Analysis (PCA), Random Forests (RF), and Support Vectors machines (SVM) [19,20] are the latest machine learning algorithm created for the learning procedure that forms the data into two classes, such as disease affected and normal [21]. However, if the dataset has been highly partial, it cannot be utilized for decision making and data analysis [22,23].

However, these classifier does not provide security to work, so the chance of prediction model exposure to outsiders is high. Nevertheless, the privacy concern associated with sensitive data is raised [24]. The concerns above include illegal sharing of confidential information, illegal usage of private data, individuals’ identification, sensitive data exposure, or inferred private information, namely disease risks from health records [25]. Therefore, data privacy, such as legal, ethical, and societal aspects, and various layered protection mechanisms must be implemented [26]. Furthermore, technological progress in recent years offers numerous ways to generate inventive techniques and algorithms [27]. This may cause technological hackers to discover information kept with less computation [28].

Nevertheless, developing new techniques does not offer superior security against hackers and cybercriminals. Therefore, cryptography-centered machine learning privacy protection has been put forward to resolve the above-mentioned issues. Several prevailing cryptography algorithms namely Elliptic Curve Cryptography and Rivests Shamir Adelman [29,30], are introduced to offer security. However, this transformation technique utilizes a certain value to multiply the original real number and then round it off to the nearby integer for attaining the transformation [31]. Nevertheless, this approximation certainly impacts the computation accuracy and damages the prediction outcomes. Therefore, one of the key intentions of this work is to introduce a relevant approach to prevent the limitation of rational numbers and ensure computation accuracy, as incorrect decision making might cause severe impact and even cause danger to patients’ life. 

Methods that preserve privacy should be developed to safeguard the privacy of medical data. Prediction models, which are created by using medical data to train them and are then used to forecast patients’ illnesses, cannot be shared with a third party since they are regarded as private and sensitive assets. Therefore, it is essential for disease prediction systems (DPS) to understand how to protect the privacy and security of prediction models. In addition to privacy and security, prediction efficiency is a crucial element that should be taken into account while constructing a disease prediction system. Learning prediction models from a huge quantity of medical data are specifically required for DPSs. 

We propose a disease prediction system based on the Elephant Herding Genetic Algorithm and Deep Learning Neural Network (EHGA-DLNN). The key features and contributions of this paper are summarized as follows:

- We propose a secure scheme for the healthcare data collected from IoT devices in modern healthcare systems.
- A Log of Round value-based Elliptic Curve Cryptography (LR-ECC) is presented for enhanced healthcare data security during the data transfer phase.
- We also propose a disease prediction system using Elephant Herding Genetic Algorithm-based Deep Learning Neural Network (EHGA-DLNN) classification algorithm.
- The proposed approach outperforms existing disease prediction systems in terms of privacy and security, according to the findings of the experiments.

Considering the above-mentioned challenges and limitations of existing approaches in DPSs, we present a novel and efficient disease prediction model by combining the LR-ECC and EHGA-DLNN algorithms. The rest of the paper is laid out as follows. Section 2 includes an overview of current privacy-preserving schemes for patients’ IoT data and a disease prediction system (DPS). Next, the detailed elucidation of the proposed work is proffered in Section 3. Finally, Section 4 exhibits the experimental outcome, and Section 5 confers the conclusion and signifies future advancements.

## 2. Related Work

Zhuoran Ma et al. [32] presented a privacy-preserving (PP) as well as a higher precision outsourced disease predictor using the random forest (RF) technique, known as PHPR. This system provided secure training with medical data belonging to several data owners and made an accurate prediction. Moreover, the raw data and computed outcomes in the rational number were safely processed and kept in the cloud without privacy leakage. Initially, privacy-preserving reckoning protocols over the rational numbers to assure the computation accuracy was designed by the system and which handled the outsourced operations immediately. Additionally, the PHPR system achieved a secure disease predictor, as demonstrated by the system. Lastly, the experimental outcomes centered on the real-world datasets established that the PHPR system not only proffers secure disease predictors over ciphertexts but also maintains the prediction accuracy as the original classifier. Nevertheless, the RF algorithm offers low efficacy.

For clinical-decision support systems centered on IoT devices, Alia Alabdulkarim et al. [33] presented a privacy-preserving single decision tree approach. To safeguard the users’ data, a homomorphic encryption cipher was utilized. Additionally, this algorithm utilizes nonces to prevent one party from decrypting the other party’s data as they would utilize identical key pairs. By 46.46%, the system outperformed the Nave Bayes method, besides the end result of the key-value and size on the run period was shown by the simulation outcomes. Moreover, the model was approved, which encountered the privacy necessity of the hospitals’ datasets, frequency of feature values, and diagnosed symptoms. However, homomorphic encryption cipher offered less security, and the system did not accept further forms of datasets.

Malathi D et al. [34] suggested a hybrid reasoning-centered Privacy-Aware Disease Prediction Support System (PDPSS). The combinative benefits of Fuzzy set theory, k-nearest neighbor, and case-centered reasoning assisted in producing improved prediction outcomes. The Disease Prediction Support System (DPSS) was extensive to the PDPSS centered on a Pailliers Homomorphic Encryption to protect patients’ sensitive details from illegal user access. The prediction system model was examined with the statistical evaluation metrics, and the experimental outcomes revealed the enhanced PDPSS’s performance in better prediction accuracy and security. The system provided satisfactory outcomes. However, the system had high communication and computational costs.

Dan Zhu et al. [35] proposed CREDO, a multi-level medical pre-diagnosis system based on multiple-label k-nearest-neighbors that was both effective and privacy-preserving (ML-kNN). The service provider (SP) first reduced the number of healthcare instances that needed to be calculated using k-means clustering and then provided service to healthcare users based on the ML-kNN classification. Before being sent out, the query vector was encrypted and directly operated in the SP; in the meantime, the medical user could only obtain the pre-diagnosis result. According to the detailed investigation, the system demonstrated that CREDO could survive a wide range of known security concerns and had a substantially lower computation complexity than the comparison system.

Xue Yang et al. [36] presented a useful and privacy-preserving system for predicting the likelihood of disease aimed at e-healthcare, called EPDP. The EPDP widely attained two stages of prediction of disease risk: the disease design training and prediction of disease whilst guaranteed privacy preservation. The super-augmenting sequence was used with a homomorphic cryptographic approach to effectively obtain the symptoms set of each disease in the disease design training phases. The bloom filter method was utilized to calculate the prediction outcomes in the stage of disease risk prediction. Moreover, wide performance evaluations established that the system achieved outstanding efficiency benefits concerning both communications and computational expenses. The system could not approve some medical practitioners; thus, accessing control in this system was hard.

Priyan Malarvizhi Kumar et al. [37] proffered an IoT and cloud-centered disease prediction and diagnosis scheme for healthcare centered on a fuzzy neural classifier. Here, the systematic approach was utilized for diabetes disease. The associated medical data were produced focused on the UCI Repositories dataset and medical sensors for predicting people affected with severe diabetes. Additionally, the system utilized a Fuzzy Rule-centered Neural Classifier to diagnose the disease and the sereneness. The experiments are held on a typical UCI repository dataset as well as the entire medical records that were gathered from several hospitals. The system’s performance was more advanced than the prevailing system in disease prediction, as indicated by the experimental outcomes. However, medical data on the cloud database were given inadequate security.

An effective and privacy-preserving disease prediction system named PPDP was proposed by C. Zhang et al. [38]. In PPDP, patients’ past medical records are encrypted and sent to a cloud server, where they can be used to train prediction models using the Single-Layer Perceptron learning method while still maintaining patient privacy. N.N. Thilakarathne et al. [39] suggested a general strategy for federated learning (FL) as a potential solution to learning about Medical IoT (MIoT) that does not necessitate moving private and sensitive data to a central cloud. In [40], a predictive approach utilizing the cloud and an IoT-based database is suggested for forecasting the diseases that used the patients’ data collected from biosensors. For the prediction, a regression technique and a classifier based on generalized fuzzy intelligence called GFIbALO were suggested. N.D. Kathamuthu et al. [41] developed a deep Q-learning-based neural network framework with a privacy preservation approach (DQ-NNPP) to safeguard sensitive patient medical data transmitted from medical IoT devices from external threats. The data confidentiality and security are less in all these models. Moreover, prediction efficiency and accuracy are also generally lacking in all the aforementioned methods. 

## 3. Methodology

**Figure 1:**
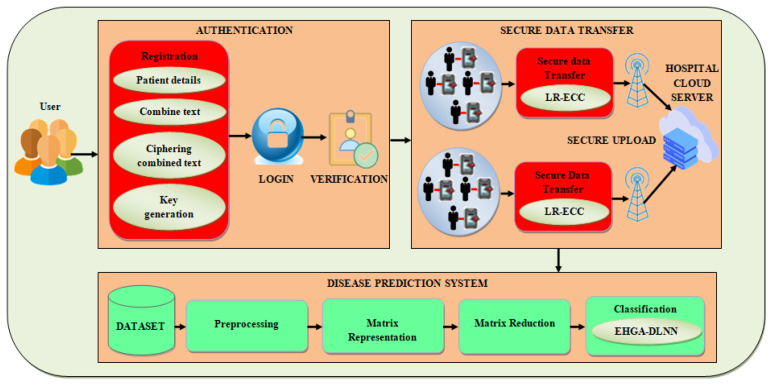
Architecture diagram of the proposed methodology.

The IoT technologies utilization in the modern healthcare application environment produces ease for patients and medical professionals as they apply to the health area. Various diseases may be decreased by performing a proactive examination of one’s health. Nevertheless, privacy concerns are increased by utilizing the patients’ disease information and medical data. The medical data’s privacy and security issues can arise owing to the delay in treatment progress, which may even jeopardize the patient’s life. Hence, it becomes a challenging problem to attain a safe disease prediction without concern for the results’ accuracy. To predict disease utilizing an efficient technique in the advanced HCS, the article proposes effective privacy preservation of the patients’ IoT data to protect the privacy of the patients’ medical data and prediction design’s security. The proposed system encompasses four segments: authentication, secure data transfer, disease prediction system, and monitoring. The IoT sensor devices have been affixed to the patient’s corpse, and afterward, the patient should register with the respective hospital utilizing the hospital’s mobile application or website. Once the login using an effective proposed authentication method is successfully done, the sensor values are sensed and are safely uploaded into the HCS through the Fog layer. Simultaneously, on the hospital side, the respective doctor can download the patient’s data safely and test these data with an earlier trained system. The proposed Privacy-Preserving Disease Prediction Model’s architecture diagram is showcased in Figure 1. 

### 3.1. Authentication Phase

To strengthen the security of the system and transmission of information, authentication is attained among doctors, healthcare staff and the Cloud Server (CS), patients and the  CS, and the healthcare center and the  CS. This stage is the first stage in the proposed system. This is an imperative step in offering access to authorized IoT sensor devices. The authentication procedure comprises three stages:

- Registration;
- Login;
- Verification.

#### 3.1.1. Registration

The administrator’s approval is required before the data can be accessed on various IoT devices connected to the healthcare system. After verification, the administrator offers data to the IoT device for authentication. The four segments comprised by this registration process are demonstrated below.

##### Patient Details

Primarily, the patient details are provided by the user in the registration stage. The patient details contain a Username, Patient Name, Sex, Age, Address, Password, Patient ID, Hospital ID, Doctor Name, and so on, that are entered by the health assistant and saved on the database. The patient details can be mathematically represented as

(1) $${\tilde{P}}_{pd}=\left\{{\tilde{p}}_{1},{\tilde{p}}_{2},{\tilde{p}}_{3},........{\tilde{p}}_{k}\right\}$$

Here, ${\tilde{P}}_{pd}$ indicates the patient details set and ${\tilde{p}}_{k\;}$ signifies the patients’ information such as sex, name, age, patient ID, etc.

##### Combine Text

After entering the patient details, merge the pair texts into single text about the concatenation procedure. Here, the user ID and the respective hospital ID are merged as a single text, which is mathematically articulated as:

(2) $${\vec{T}}_{ct}^{\prime \prime }={\tilde{p}}_{u}⊕{\tilde{\;p}}_{h}$$

Here, ${\vec{T}}_{ct}^{\prime \prime }$ signifies the combined text, ${\tilde{p}}_{u}\;$ along with ${\tilde{p}}_{h}$ illustrates the user ID and hospital ID correspondingly that is extracted from ${\tilde{P}}_{pd}$.

##### Ciphering Combined Text

The ciphering procedure is executed after completing the concatenation of the text as above during the registration to convert as ciphertext wielding the substitution cipher. A substitution cipher is an encrypting method in which plaintext units are changed by ciphertext in accordance with a preset system. Those where the cipher alphabet is just the plaintext alphabet’s cyclical shift are the simplest among all the substitution ciphers. These variant ciphertext values offer better security. Furthermore, the alphabets’ plaintext elements can be stretched easily, including common syllables, punctuation, numbers, and the fundamental twenty-six letters. Mathematically, letter encryption can be expressed as:

(3) $${\left({\vec{T}}_{ct}^{\prime \prime }\right)}_{encrypt}=\left({\vec{T}}_{ct}^{\prime \prime }\right)\;mod\;26$$

Here, ${\left({\vec{T}}_{ct}^{\prime \prime }\right)}_{encrypt}\;$ denotes the ciphertext of the combined text. At the time of verification, these ciphertexts are sent to the data owner by the CS. The CS inquires the user to transfer the ciphertext if a user endeavors to download any file present in the CS. While the right ciphertext is sent by the user, the CS validates the user as an authorized user and the user is permitted to access the data. The substitution cipher is mathematically articulated as:

(4) $${\left({\vec{T}}_{ct}^{\prime \prime }\right)}_{encrypt}\overset{Matched}{\to }\left(CS\overset{confirms}{\to }{\tilde{A}}_{au}\right)$$

(5) $${\left({\vec{T}}_{ct}^{\prime \prime }\right)}_{encrypt}\overset{Not\;\;Matched}{\to }\left(CS\overset{confirms}{\to }{\tilde{A}}_{ur}\right)$$

Here, ${\tilde{A}}_{au}$ along with ${\tilde{A}}_{ur}$ represents the authorized and unauthorized users correspondingly.

##### Key Generation

Here, the Cloud will create the public key as well as the private key. The public key has been presented; however, the private key is sent to the user’s email that is offered during the registration time; this declares that data encryption is done, and the keys are produced. The Cloud provider will inquire for the private key as quickly as the user requests to view files. If the user offers the correct private key, the decryption of the text file is executed via the cloud provider and depicted it to the user. It showcases only the encrypted data format, not the original file, when the private key is incorrect. The mathematical expression of the public key, together with the private keys produced by the cloud, is:

(6) $$CS\overset{\left({\overset{↔}{K}}_{pu}^{\prime \prime },\;{\overset{↔}{K}}_{pr}^{\prime \prime }\right)}{\to }User$$

Here, ${\overset{↔}{K}}_{pu}^{\prime \prime }$ signifies the public key as well as ${\overset{↔}{K}}_{pr}^{\prime \prime }$ represents the private key. The secret key has been computed to improve the security level. The secret key was calculated by considering the round log value of the ${\overset{↔}{K}}_{pu}^{\prime \prime }\;$ along with the ${\overset{↔}{K}}_{pr}^{\prime \prime }$, which is mathematically articulated as:

(7) $${\overset{↔}{K}}_{se}^{\prime \prime }=log\;\left({\overset{↔}{K}}_{pu}^{\prime \prime }\;⊕\;{\overset{↔}{K}}_{pr}^{\prime \prime }\right)$$

Here, ${\overset{↔}{K}}_{se}^{\prime \prime }$ signifies the secret key and ⊕  stands for the round log value of the ${\overset{↔}{K}}_{pu}^{\prime \prime }\;$ as well as the ${\overset{↔}{K}}_{pr}^{\prime \prime }$.

#### 3.1.2. Login

Login is a credential set wielded for validating a user. Mostly, they comprise the username as well as the password. The login segment lets a user for getting accessibility to an application via entering their username and password. The patients ought to input the authentication data offered for authentication using the administrator while logging in to the system. The patient should enter the user-id, password, and ciphertext while logging in.

#### 3.1.3. Verification

The verification procedure has been carried out after the system is logged in. The system would match this segment’s user-id, username, password, and ciphertext. The system finalizes that the patient is already registered with the respective Hospital Cloud Server if all the details are matched. Or else the system returns to the registration stage. 

### 3.2. Secure Data Transfer Phase

Here, the IoT-sensed data are encrypted utilizing the Left to Right Elliptic Curve Cryptography (LR-ECC) and is sent into the CS through the Fog Layer. ECC is a key-centered method for encrypting data. ECC concentrates on public and private key pairs to decrypt and encrypt web traffic. For the security level enhancement, the secret key is computed that has been examined earlier in the key generation segment during registration. The user encrypts his/her file (${\tilde{C}}_{tx}$) after login successfully utilizing the sender’s private key, the receiver’s public key, and the secret key that generates the ciphertext, which is expressed as:

(8) $$E\left({\tilde{C}}_{tx}\right)={\tilde{C}}_{tx}+\left(Rn*{\overset{↔}{K}}_{pr}^{\prime \prime }\;{\overset{↔}{F}}_{pu}^{\prime \prime }\right)\;*K_{se}^{\prime \prime }$$

Here, $E\;\left({\tilde{C}}_{tx}\right)$ denotes the ciphertext, ${\overset{↔}{K}}_{pr}^{\prime \prime }\;$ signifies the user private key, ${\overset{↔}{F}}_{pu}^{\prime \prime }$ denotes the receiver public key, ${\overset{↔}{K}}_{se}^{\prime \prime }$ represents the secret key, along with Rn denotes a random number in the range (1, *n* − 1). This ciphertext is sent over the cloud. It decrypts the ciphertext utilizing the coalition of the sender’s public key, the receiver’s private key, and the secret key to recover the plain text. In contrast, the receiver is the expected recipient (explicitly) the doctor. It is articulated as:

(9) $$\tilde{C}{\;}_{tx}=\frac{\left(E\left(\tilde{C}{\;}_{tx}\right)-{\overset{↔}{K}}_{pu}^{\prime \prime }\;{\overset{↔}{F}}_{pr}^{\prime \prime }\right)}{{\overset{↔}{K}}_{se}^{\prime \prime }}$$

Here, ${\tilde{C}}_{tx}$ signifies the original plain text, ${\overset{↔}{K}}_{pu}^{\prime \prime }$ as well as ${\overset{↔}{F}}_{pr}^{\prime \prime }$ signifies the sender’s public key and the receiver’s private key correspondingly.

### 3.3. Disease Prediction System (DPS)

In the proposed system, the disease prediction system (DPS) is the main process that predicts the chance of a disease’s presence in a patient centered on their symptoms. The sensed values should be tested to find if the patient comprises the disease. First, train the disease dataset, before the values are tested. The training system has four phases: data collection, preprocessing, matrix representation, matrix reduction, and classification, which are described in the below section. 

#### 3.3.1. Data Collection

The primary procedure is the collection of the disease data set (Hungarian dataset). Then, the description of such a dataset is represented as:

(10) $$H_{ds}^{\prime \prime }=\left\{h_{1},h_{2},h_{3},...........h_{m}\right\}$$

Here, $H_{ds}^{\prime \prime }$ stands for the disease dataset for additional processing, $h_{m}$ signifies the m—number of dataset’s information.

#### 3.3.2. Preprocessing

The dataset preprocessing is the training system’s primary process, which is vital in all processes since it directly affects the system’s success rate. Since the real-world data are unclean, this decreases the data’s complexity under examination to initially execute the preprocessing. The dataset can contain duplicate data. This step evades the training of the same information repeatedly for removing the redundant data via the execution of data de-duplication.

##### Data Deduplication

Data deduplication is among the methods that permit cloud users to efficiently administer their cloud storage space by avoiding repeated data storage and saving bandwidth. It evades the repeated training of duplicate data. 

Data deduplication contains two phases: the missing value imputations, as well as the min-max normalization, which is detailed as:

- Missing value imputation

The method of changing the missing data into substituted values is termed Imputation. The dataset contains variables in which few data are missing. While any records encompass missing values in their records, these values can be loaded via the changing of missing values aimed at a specific attribute with the average value aimed at that attribute. Here, the missing value is denoted as ‘?’.

- MinMax normalization

The method of decomposing tables for eliminating the data redundancy (or) repetition and undesirable features, namely insertion, updating together with deletion anomalies, is labeled as Normalization. Minimum–Maximum Normalization technique assembles data aiming at more effective access. The system produces efficient outputs while normalization has been implemented. This technique operates by altering the data values in a particular range, namely amid 0 to 1 or amid −1 to 1 utilizing the minimal and maximal values. Subtract $\min\left(H_{ds}^{\prime \prime }\right)$ from every single data to execute this. It is articulated as

(11) $$H_{ds}^{\prime \prime }-\min(H_{ds}^{\prime \prime })$$

After that, change the data to comprise 1 as the upper bound. Divide every value by the original gamut to perform this. It is expressed as,

(12) $$\frac{H_{ds}^{\prime \prime }}{\max(H_{ds}^{\prime \prime })-\min(H_{ds}^{\prime \prime })}$$

Lastly, combining Equation (11) as well as Equation (12) obtains the normalized value, that is,

(13) $$MinMax=\frac{H_{ds}^{\prime \prime }-\min(H_{ds}^{\prime \prime })}{\max(H_{ds}^{\prime \prime })-\min(H_{ds}^{\prime \prime })}$$

The missing values are replaced centered on the above-given procedures via the minimal and maximal values and efficiently enhance the data integrity.

#### 3.3.3. Matrix Representation

Next, the preprocessed data are regarded as a matrix representation. Commonly, the preprocessed data have been signified as a L × P matrix, in which L signifies the number of instances as well as P signifies the number of attributes, namely sex, age, address, et cetera, present in the experiment. Every cell in the matrix is equated as

(14) $$M_{rep}^{\prime \prime }=\left\{\begin{array}{c} I_{1,1}\;\;I_{1,2}\;\;\ldots \;\;I_{1,n}\\ I_{2,1}\;\;I_{2,2}\;\ldots \;\;I_{2,n}\\ I_{n,1}\;\;I_{n,2}\;\ldots \;\;I_{n,n} \end{array}\right\}$$

Here, $M_{rep}^{\prime \prime }\;$ denotes the preprocessed data’s matrix representation.

#### 3.3.4. Matrix Reduction

Matrix reduction is the data conversion of a higher dimensional space to lower-dimensional spaces so that the low dimensional illustration retains a few noteworthy properties of the data (original). Gaussian Kernels-centered Linear Discriminants Analysis (GK-LDA) algorithm is utilized to decrease the preprocessed data matrix. LDA is among the renowned supervised techniques implemented in various high-dimension reduction processes. It encrypts biased information by detecting directions that reduce the betwixt-class scatter to within-class scatter ratio. While the total samples are small when contrasted to the samples’ dimensionality and attain low reduction accuracy, Small-Sample-Size (SSS) issue occurs in LDA. This issue can be solved by utilizing a Gaussians Kernel function included in the existent LDA to ameliorate the reduction accuracy. 

The GK-LDA’s algorithmic methods are described below.

Step 1: First, take the preprocessed data’s matrix representation as $M_{rep}^{\prime \prime }$
Step 2: Next, take ${\tilde{B}}_{c}\;$ and ${\tilde{W}}_{c}$ that represents the betwixt-class as well as within-class scatter matrices that are articulated as:

(15) $${\tilde{B}}_{c}=\overset{a}{\underset{m=1}{\sum }}s_{m}\left({\left(M_{rep}^{\prime \prime }\right)}_{m}-\left(M_{rep}^{\prime \prime }\right)\right)\;{\left({\left(M_{rep}^{\prime \prime }\right)}_{m}-\left(M_{rep}^{\prime \prime }\right)\right)}^{T}$$

(16) $${\tilde{W}}_{c}=\overset{a}{\underset{m=1}{\sum }}\left(\overset{q_{m}}{\underset{n=1}{\sum }}\left(D_{n}-{\left(M_{rep}^{\prime \prime }\right)}_{m}\right)\;{\left(D_{n}-{\left(M_{rep}^{\prime \prime }\right)}_{m}\right)}^{T}\right)$$

Here,

(17) $${\left(M_{rep}^{\prime \prime }\right)}_{m}=\frac{1}{s_{m}}\;\underset{D_{n}\in D_{m}}{\sum }D_{n}$$

(18) $$\left(M_{rep}^{\prime \prime }\right)=\frac{1}{s}\;\overset{a}{\underset{m=1}{\sum }}\underset{D_{n}\in D_{m}}{\sum }D_{n}$$

Step 3: For the reduction accuracy level enhancement, the Gaussians kernel is utilized for computing the distances among the data points, in addition to the Kernel matrix is gauged (with the kernel trick), which is articulated as:

(19) $$\kappa \;\left(D_{m},D_{n}\right)=\exp\;\left(-\gamma_{mn}\;|\left|D_{m}-D_{n}\right|{|}^{2}\right)$$

Here, it signifies the weight determined by the Gaussian kernel.

Step 4: LDA searches for a linear subspace R (c−1  components) within which the projections of the disparate classes are best divided, as stated using maximizing the subsequent discriminant criterion.

(20) $$V\left(R\right)=\max\frac{ToM\;\left\{R^{T}\;{\tilde{B}}_{c}\;R\right\}}{ToM\;\left\{R^{T}\;{\tilde{W}}_{c}\;R\right\}}$$

where ToM (.) signifies the trace of matrix. In addition to the orthogonal constraint of R, this can well be resolved as a generalized eigen-vector along with the eigenvalue issue stated below:

(21) $${\tilde{B}}_{c}\;R_{m}=\lambda_{m}\;{\tilde{W}}_{c}\;R_{m}$$

where $R_{m}$ and $\lambda_{m}$ signifies the *m*-th generalized eigenvector and eigenvalue of ${\tilde{B}}_{c}\;$ concerning ${\tilde{W}}_{c}$.

Step 5: Order the eigenvectors by means of lessening the eigenvalue. Finally, the reduced feature set can well be attained by,

(22) $${\left(H_{rf}\right)}_{v}={\left(M_{rep}^{\prime \prime }\right)}_{m}\cdot \;R_{m}$$

where ${\left(H_{rf}\right)}_{v}$ signifies the reduced matrix set that is generated as a linear combination of the entire inputted matrix depiction of the preprocessed data $\left(M_{rep}^{\prime \prime }\right)$. 

#### 3.3.5. Classification Using Elephant Herding Genetic Algorithm Based Deep Learning Neural Network (EHGA-DLNN)

Lastly, the ${\left(H_{rf}\right)}_{v}$ is inputted to the classifier. Classification is the main element principally utilized for training the data to make the disease prediction, which is done by Elephant Herding Genetic Algorithm based Deep Learning Neural Network (EHGA-DLNN). This algorithm trains the dataset for better classification. Compared to other machine learning algorithms, typical deep learning algorithms can produce new features from a limited number of features in the training dataset. Compared to other machine learning algorithms, typical deep learning algorithms can produce new features from a limited number of features in the training dataset. Normal DLNN gives satisfactory results, but less accuracy is produced by the random Weight Values (WV) to classify normal and disease-affected severity. The Elephant Herding with Genetic Algorithm (GA) can be employed to optimize the WV to reduce the backpropagation problem in the DLNN algorithm. The GA steps, namely Crossover and Mutation (CM), were hybrid with the updation step in the sandpiper algorithm to ameliorate the search accuracy. The Hidden Layer (HL), the input layer, and the output layer are the three layers of DLNN. These layers, together with the algorithmic method, are explained below.

- The Input Layer

This is the primary layer, which accepts inputted values and transmits them to the succeeding layer. Initially, the ${\left(H_{rf}\right)}_{v}$ of preprocessed data is assigned for training the system, and their equivalent weight is also ascertained, which is described as:

(23) $${\left(H_{rf}\right)}_{v}=\left\{h_{1},h_{2},h_{3},..........h_{k}\right\}$$

(24) $${\left(W_{ew}\right)}_{v}=\left\{w_{1},w_{2},w_{3},...........w_{k}\right\}$$

herein, the arbitrary WV is ineffective in accurately predicting customer purchase intention. Thus, EHGA optimizes these WVs. 

The Elephant Herding Optimizations (EHO) is a comparatively novel population-centered optimization technique. It imitates the herding behavior and can well be designed into ‘2’ operators: clan updating and separating operators. The EHGA procedures are elucidated as:

Initially, the population space, belief space, and adjustable operator are initialized. Then, when it comes to the clan updating, the elephants’ positions are updated via the solution search strategy as follows:

(25) $$\;T_{new,xl}^{k}=T_{xl}^{l}+\alpha *\left(T_{best,xl}-T_{xl}^{l}\right)*g$$

wherein, $T_{new,xl}^{k}\;$ and $T_{xl}^{l}$ implies the new and old position of the elephant k   on the clan xl  and $T_{est,xl}\;$ signifies the matriarch of clan xl, g implies the arbitrary number generated in the gamut [0, 1] as well as α signifies the scale factor that ascertains the matriarch’s effect. Following the clan update procedure, the worst elephants in the clans are eliminated, and their new places in the search space are generated arbitrarily using a separation operator. Following the clan update procedure, the worst elephants in the clans are eliminated, and their new places in the search space are generated arbitrarily using a separation operator and it is expressed as:

(26) $$\;T_{worst,xl}=T{\left(Tmin_{max}\times f_{r}\right)}_{min}$$

where $T_{min}\;$ and $T_{max}$ signifies the upper and lower bounds on the search space, $f_{r}$ implies the number ascertained arbitrarily in the gamut [0, 1] and $T_{worst,xl}\;$ implies the male elephants with the worst fitness value on the clan xl. Before the newly updated position, the proposed work hybrid GA with this EHO algorithm provides the optimal solution. Here, the GA steps, say CM, were hybrid with the updating step to ameliorate the search accuracy. Therefore, the equation above utilizes CM operation before updating a new position. Here, the two-point crossover is utilized and is described as

(27) $$T\;\left(t+1\right)=T_{f_{r}}\left(t\right)⊕\;C_{1}⊕\;C_{2}$$

(28) $$C_{1}=\frac{\left|T\;\left(t\right)\right|}{3}$$

(29) $$C_{2}=C_{1}+\frac{\left|T\;\left(t\right)\right|}{2}$$

This makes task scheduling more effective. Here, t indicates the iteration level, and $C_{1}$ indicates the two points that are chosen as points of crossover. Subsequently, the mutation is done using placing the new genes rather than the genes on every chromosome. The replacement genes are sporadically created genes with no chromosomal duplication. After that, the new position (i.e., the optimal weight, ${\left(O_{w}^{\prime \prime }\right)}_{v}$) is updated using the sandpiper algorithm. Mathematically, it is expressed as:

(30) $${\left(O_{w}^{\prime \prime }\right)}_{v}=T\left(t+1\right)*T_{bset,xl}$$

After that, the inputted value (converted questionnaire data) is multiplied by the ${\left(O_{w}^{\prime \prime }\right)}_{v}$ that is arbitrarily chosen and then totally summed up. It is stated as:

(31) $${\tilde{e}}_{v}=\overset{n}{\underset{v=1}{\sum }}\left({\left(H_{rf}\right)}_{v}\cdot \;{\left(O_{w}^{\prime \prime }\right)}_{v}\right)$$

where $\tilde{e}$ signifies the assigned value. Next, the network’s activation function (AF) is calculated, which is exhibited as:

(32) $${\overset{↔}{A}}_{v}=f\left(\overset{n}{\underset{v=1}{\sum }}{\left(H_{rf}\right)}_{v}\cdot \;{\left(O_{w}^{\prime \prime }\right)}_{v}\right)$$

where ${\overset{↔}{A}}_{v}\;$ signifies the AF that is inputted to the HL.

- The Hidden Layer

In this HL, the network multiplied the AF’s output with the WVs and then summed it up with the bias value. Mathematically, it is exhibited as:

(33) $${\overset{↔}{H}}_{v}=Bias+\overset{n}{\underset{v=1}{\sum }}{\overset{↔}{A}}_{v}\cdot \;{\left(O_{w}^{\prime \prime }\right)}_{v}$$

where ${\overset{↔}{H}}_{v}\;$ signifies HL’s output and  Bias  implies the bias value.

- The Output Layer

This is accountable for generating the last outcome. It is stated as:

(34) $${\overset{↔}{O}}_{v}=Bias+\overset{n}{\underset{v=1}{\sum }}{\overset{↔}{H}}_{v}\cdot \;{\left(O_{w}^{\prime \prime }\right)}_{v}$$

Lastly, the loss function is calculated using the following equation:

(35) $$Loss_{v}=\left[{\overset{↔}{G}}_{v}+{\overset{↔}{O}}_{v}\right]$$

where ${\overset{↔}{G}}_{v}\;$ signifies the desired outcome of the neural network. In this case, the loss function’s threshold is set to the smallest value. If the initialized threshold value meets this fitness, the output is displayed as the last output. If the initialized threshold value does not match this fitness, the WV’s position is reissued, and the same EHGA optimizes the WV. The output unit is determined again using this EHGA-DLNN technique, and the output data are trained for retrieval. The EHGA-DLNN pseudocode is elucidated in Algorithm 1.
**Algorithm 1** EHGA-DLNN algorithm        **Input:** Reduced matrix set ${\left(H_{\mathrm{rf}}\right)}_{v}$
        **Output:** Classified disease-affected data.           $\mathrm{Initialize}\;{\left(W_{\mathrm{ew}}\right)}_{v}$, Bias$,\;{\;A\;↔}_{v}\;$ $\mathrm{and}\;{\;H\;↔}_{v}$           **Calculate** the number of training samples                 NumData=λ
             **if**
(λ=0)                 Error (λ is not an integer)            **end if**            **for** each reduced data do                  **Update** the position of the weight value using EHGA                  **Update** the new position using,${\left(O_{w}^{\prime \prime }\right)}_{v}=T\left(t+1\right){*T}_{\mathrm{bset},\mathrm{xl}}$                   **while** (*v < iter*) do                        **Perform** activation function by using$\;{\overset{↔}{\;A}}_{v}=f\left(\overset{n}{\underset{v=1}{\sum }}{\left(H_{\mathrm{rf}}\right)}_{v}\cdot \;{\left(O_{w}^{\prime \prime }\right)}_{v}\right)$                              //calculation of activation function                         **for**${\overset{↔}{H}}_{v}$ do                                **Calculate** hidden layer output by                                  ${\overset{↔}{H}}_{v}=\mathrm{Bias}+\overset{n}{\underset{v=1}{\sum }}{\overset{↔}{A}}_{v}\cdot \;{\left(O_{w}^{\prime \prime }\right)}_{v}$
                                **Compute** output layer output by       ${\overset{↔}{O}}_{v}=\mathrm{Bias}+\overset{n}{\underset{v=1}{\sum }}{\overset{↔}{H}}_{v}\cdot \;{\left(O_{w}^{\prime \prime }\right)}_{v}$
                          **end for**
                     **end while**


### 3.4. Monitoring

The corresponding doctor on the hospital side can download patient data securely and test these data with the already trained system (DPS).

## 4. Results and Discussion

The vital task in this work is the data classification which classifies the data as normal or disease-affected with severity. At the exact time, security is crucial for securely transferring the data. In JAVA, this system is executed. The experiments were done utilizing the medical datasets to examine the proposed work concerning the parameters, namely specificity, f-measure, recall, accuracy, precision, and sensitivity. Likewise, the proposed system’s security is computed regarding security level analysis, decryption time, and encryption time. The proposed system’s security is analogized with the prevailing methodology initially. Utilizing the disparate classification algorithm, various experiments are done on classifying the disease affected. The experimental outcome is also tested to evaluate the proposed technique’s performance.

### 4.1. Evaluation Parameters

The proposed system considered several standard assessment parameters: encryption time, decryption time, f-measure, accuracy, recall, precision, sensitivity, and specificity. These metrics’ brief descriptions are elucidated below.

(i) Encryption time: It is the difference between the encryption starting and ending times and the time taken by the encryption algorithm to construct a ciphertext from plain text.
(ii) Decryption time: The difference between the encryption beginning and finishing times is used to calculate it.
(iii) Accuracy: It might be indicated by the probability that a record is precisely identified that it could be normal or disease affected.
(iv) Sensitivity: The rate of correct differentiation between normal and disease-affected data.
(v) Specificity: It is the rate of accurate classification of disease that affects the total classified results.
(vi) Precision: For a certain class, it is the count of accurately envisaged records over the entire envisaged records.
(vii) Recall: For a specific class, it is the count of accurately envisaged disease-affected outcomes over all the records available in the dataset.
(viii) F-measure: It utilizes precision and recall for the holistic estimation of a model and is described as their harmonic mean.

### 4.2. Analysis of Security Level Performance

**Figure 2:**
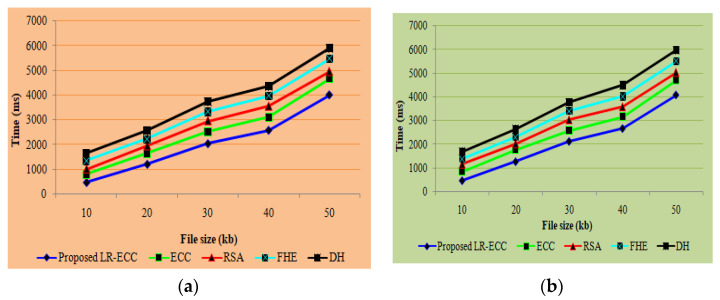
(**a**) Encryption time and (**b**) decryption time graph for the proposed method.

Here, regarding the security level analysis, encryption time, and decryption time, the performance of the new LR-ECC method is compared to that of the existing ECC, RSA, Fully Homographic Encryption (FHE), and Diffie Hellman (DH) algorithms. A metrics-based performance comparison is elucidated in Figure 2.

Regarding the encryption time and decryption time, Figure 2 delineates the proposed LR-ECC’s performance when analogized with the conventional ECC, RSA, FHE, and DH algorithms. The file sizes range from 10 kb to 50 kb. Figure 2a shows that the proposed approach takes 465 milliseconds to encrypt a 10 kb file. In contrast, the existing ECC, RSA, FHE, and DH algorithms take 801 milliseconds, 1013 milliseconds, 1346 milliseconds, and 1646 milliseconds, respectively, to encrypt the data. Similarly, the proposed one achieves more excellent performance for the 20 to 50 kb file size. Figure 2b reveals that for the file size of 10 kb, the proposed technique takes 475 ms to decrypt the file. In contrast, the prevailing proffers lower performance than the proposed one, and the proposed one requires 1275 ms time to decrypt the data for file size 20 kb, which is also less than the prevailing methodology. The discussion generally reveals that the proposed one has top-level performance when analogized with the prevalent methods.

**Figure 3:**
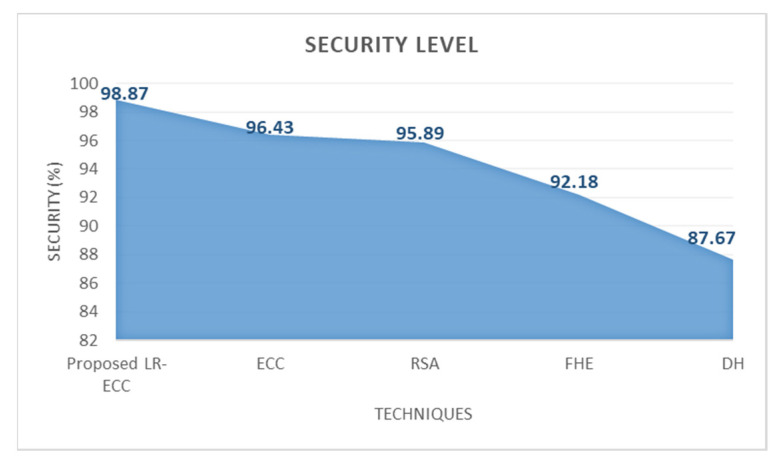
Security level analysis of the proposed LR-ECC methodology.

Concerning the proposed system’s security level analysis, Figure 3 outlines the proposed LR-ECC’s performance with the traditional ECC, RSA, FHE, and DH algorithms. The conventional DH algorithm provides 87.67% security when analogized with the proposed one, which is significantly less. Moreover, the prevailing ECC, RSA, and FHE algorithm offers 96.43%, 95.89%, and 92.18%, which is also less than the proposed one, but the proposed LR-ECC offers the top-level security of 98.87%. Therefore, the discussion indicates that high performance is attained by the proposed one when analogized with all the existing methodologies.

### 4.3. Performance Analysis of Classification

By utilizing various classification algorithms, say ANN, DLNN, KNN, SVM, and the proposed EHGA-DLNN, several experiments have been performed for the classification of the disease affected. Here, a performance comparison is accomplished by employing various performance metrics, such as precision, specificity, accuracy, F-measure, sensitivity, and recall. The performance examination of these metrics is verified in Table 1.

About various qualitative performance metrics, namely precision, specificity, accuracy, f-measure, sensitivity, and recall, the above table delineates the proposed EHGA-DLNN’s performance with that of the traditional DLNN, ANN, KNN, and SVM classifier. The table reveals that the prevailing SVM classifier proffers low-level performance than the proposed EHGA-DLNN classifier. Moreover, the prevailing DLNN, ANN, and KNN algorithms offer less performance when analogized with the proposed classifiers, but 95.32% precision, 96.36% specificity, 98.35% accuracy, 96.37% f-measure, 97.33% sensitivity, and 96.69% recall are offered by the EHGA-DLNN algorithm. This is a high score compared to all of the existing classifiers. Therefore, the proposed EHGA-DLNN could predict the disease faster with higher accuracy, which could be inferred from the outcomes. Additionally, it is described and elucidated in the below figures.

**Figure 4:**
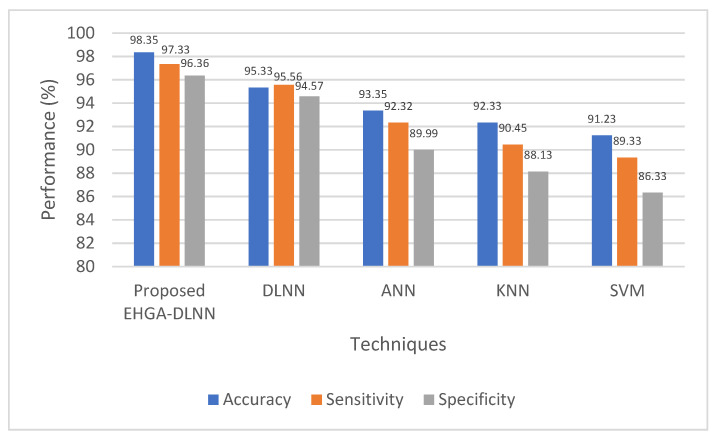
Comparative analysis of the proposed methodology with the existent methodologies.

By analogizing the proposed EHGA-DLNN technique with the prevailing DLNN, ANN, KNN, and SVM techniques, Figure 4 displays the achieved accuracy, sensitivity, and specificity values. In this case, the proposed methodology outperforms all existing approaches. Regarding the accuracy metric, the proposed EHGA-DLNN classifier proffers 98.35 % accuracy. In contrast, the prevailing DLNN, ANN, KNN, and SVM classifiers offer accuracy of 95.33%, 93.35%, 92.33%, and 91.23%, respectively, less analogized with the proposed one. Likewise, the specificity of 96.36% and sensitivity of 97.33% are achieved by the EHGA-DLNN classifier. Thus, it is inferred that the EHGA-DLNN achieves better accuracy when analogized with the prevailing system.

**Figure 5:**
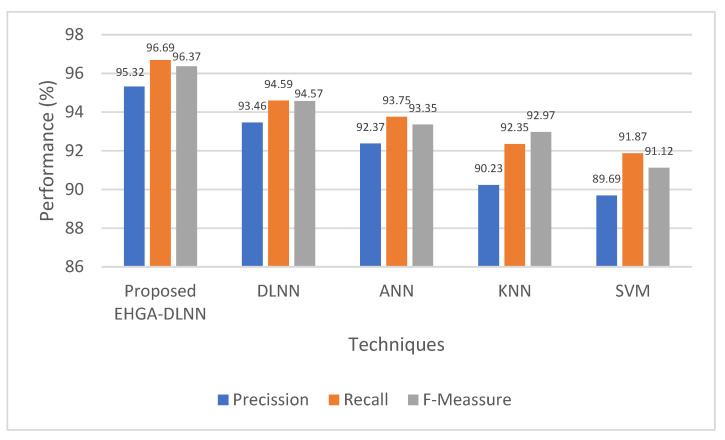
Precision, recall, and F-measure graph for the proposed methodology.

The proposed EHGA-DLNN’s performance with various traditional algorithms, namely DLNN, ANN, KNN, and SVM algorithms, are exhibited in Figure 5. The performance analysis is done using various qualitative metrics, such as F-measure, precision, and recall. This system offers high F-measure, precision, and recall value, which is revealed by the disease prediction analysis. However, 91.12% f-measure, 89.69% precision, and 91.87% recall are offered by the prevailing SVM, which is less when analogized with the proposed method. In contrast, the proposed one attains 96.37% f-measure, 95.32% precision, and 96.69% recall. Thus, regarding the f-measure, precision, and recall metrics, the achieved outcomes approve that the EHGA-DLNN classifier can better predict the disease severity than the prevailing methodologies.

## 5. Conclusions

The patients’ privacy and security of sensitive healthcare data are put at risk in modern disease prediction systems that use the medical IoT devices. In the context of this current healthcare system, this proposed work provided an effective method for protecting patient privacy when using IoT healthcare data to predict diseases. We employed a novel approach of combining LR-ECC and EHGA-DLNN techniques in our DPS. Performance analysis of secure data transmission and performance analysis of classification were both used to compare the system’s performance. The proposed LR-ECC’s performance is initially evaluated against the conventional ECC, RSA, FHE, and DH algorithms regarding encryption time, decryption time, and security level analysis. The proposed technique has a security rating of 98.87%. Subsequently, the proposed EHGA-DLNN’s performance is weighed against the existent DLNN, ANN, KNN, and SVM, and it attained an accuracy of 98.35 %, which is more significant when contrasted with the prevailing classifiers. The experimental outcome displays the proposed work’s performance is superior to that of the prevailing systems for disease prediction and provides better privacy and security. This model can be improved in the future with more generic strategies so that it can accept additional dataset types while maintaining greater security and privacy.

## Figures and Tables

**Table 1:** The proposed method’s performance with the existing approaches.

Metrics	Proposed EHGA-DLNN	DLNN	ANN	KNN	SVM
Accuracy	98.35	95.33	93.35	92.33	91.23
Sensitivity	97.33	95.56	92.32	90.45	89.33
Specificity	96.36	94.57	89.99	88.13	86.33
Precision	95.32	93.46	92.37	90.23	89.69
Recall	96.69	94.59	93.75	92.35	91.87
F-measure	96.37	94.57	93.35	92.97	91.12
